# Evidence of Antiproliferative Activity in the Liverwort *Isotachis serrulata* from Southern Ecuador

**DOI:** 10.3390/molecules31071208

**Published:** 2026-04-06

**Authors:** José Miguel Andrade, Ángel Benítez, Aday González-Bakker, Luis Cartuche, José M. Padrón, Ana R. Díaz-Marrero, José J. Fernandez

**Affiliations:** 1 Departamento de Química, Universidad Técnica Particular de Loja (UTPL), Calle Paris s/n y Praga, Loja 110107, Ecuador; 2 Instituto Universitario de Bio-Orgánica Antonio González (IUBO AG), Universidad de La Laguna (ULL), 38206 La Laguna, Tenerife, Spain; agonzaba@ull.es (A.G.-B.); jmpadron@ull.es (J.M.P.); 3 Biodiversidad de Ecosistemas Tropicales-BIETROP, Herbario HUTPL, Departamento de Ciencias Biológicas y Agropecuarias, Universidad Técnica Particular de Loja (UTPL), Calle Paris s/n y Praga, Loja 1101607, Ecuador; arbenitez@utpl.edu.ec; 4 Facultad de Ciencias Agropecuarias, Universidad Técnica de Machala, 5.5 km Pan-American Av., Machala 070150, Ecuador; lcartuche1@utmachala.edu.ec; 5 Instituto de Productos Naturales y Agrobiología (IPNA), Consejo Superior de Investigaciones Científicas (CSIC), Avenida Astrofísico Francisco Sánchez 3, 38206 La Laguna, Tenerife, Spain; 6 Biotecnología Marina, IUBO-ULL, Unidad Asociada al IPNA-CSIC, 38206 La Laguna, Tenerife, Spain; 7 Departamento de Química Orgánica, Universidad de La Laguna (ULL), 38206 La Laguna, Tenerife, Spain

**Keywords:** *Isotachis*, foliose liverwort, distribution models, antiproliferative

## Abstract

Natural products from bryophytes represent an underexplored source of structurally diverse bioactive compounds. In this study, extracts of *Isotachis serrulata* collected in southern Ecuador were evaluated for antiproliferative activity against five human tumor cell lines. Sequential extraction and chromatographic fractionation yielded six fractions, among which fraction IsF5 displayed the most notable activity, particularly against lung (SW1573) and breast (T-47D) cancer cell lines, with GI_50_ values within the moderate activity range according to National Cancer Institute criteria. Phytochemical investigation of IsF5 revealed the presence of two glycosylated aromatic constituents, tentatively assigned as tachioside and isotachioside, based on comparative ^1^H and ^13^C NMR spectroscopic analysis. These compounds were obtained as a mixture and were not evaluated individually due to limited material. Additionally, species distribution modeling using MaxEnt indicated that *I. serrulata* is primarily associated with humid montane and páramo ecosystems in the southern, central and northern Andes of Ecuador, where elevation and precipitation variables strongly influence its distribution. This study provides the first integrated assessment of the antiproliferative activity, chemical profiling, and ecological distribution of *I. serrulata*.

## 1. Introduction

Natural products remain a major source of bioactive molecules in drug discovery, with many approved drugs and lead compounds derived from natural scaffolds [1,2,3]. However, several taxonomic groups remain poorly explored, limiting the discovery of structurally unique metabolites with potential therapeutic relevance [4]. Among these, bryophytes—comprising mosses, liverworts, and hornworts—are an early-diverging lineage with distinctive ecological adaptations and metabolic capabilities, yet they remain comparatively understudied from phytochemical and pharmacological perspectives despite their wide distribution and ecological significance [5,6].

Among bryophytes, liverworts (Marchantiophyta) are recognized for producing structurally distinctive secondary metabolites, including terpenoids, bibenzyls, bisbibenzyls, and phenolic derivatives that are rarely encountered in higher plants [7,8,9]. Numerous metabolites isolated from liverworts have demonstrated relevant biological activities, including antimicrobial, cytotoxic, antiproliferative, and anti-inflammatory effects [10,11,12]. More recent comprehensive surveys further document the structural diversity and pharmacological potential of liverwort secondary metabolites across multiple genera [7,13].

In contrast, the genus *Isotachis* (Balantiopsidaceae), distributed in tropical and subtropical regions including Andean ecosystems of South America [14,15], has received comparatively little attention. Available reports are largely limited to the identification of isolated metabolites, and systematic pharmacological evaluations of *Isotachis* species remain scarce [16]. Phytochemical studies of the genus *Isotachis* have reported benzyl cinnamate and benzyl benzoate, as well as phenyl and β-phenylethyl cinnamates [17,18].

In parallel with phytochemical research, species distribution modeling has become an important tool for understanding the ecological and geographical patterns of plant species, particularly in heterogeneous and biodiverse landscapes [19,20]. Such models allow the identification of suitable habitats and provide insights into the environmental factors influencing species occurrence [21]. When integrated with chemical and biological studies, distribution analyses can contribute to a more comprehensive understanding of species selection and potential metabolic variability.

In this context, the present study aims to provide the first integrated evaluation of the antiproliferative potential of *I. serrulata*, using the GI_50_ parameter (µg/mL) to assess growth inhibition in human cancer cell lines, together with a chemical investigation of its extracts and the chemical profiling and tentative structural assignment of selected secondary metabolites. Additionally, a species distribution model of *I. serrulata* across southern Ecuador is presented to support the ecological relevance of the species. As far as we are aware, this is the first report combining antiproliferative activity, chemical characterization, and distribution modeling for *I. serrulata*.

## 2. Results and Discussion

Dried material of *I. serrulata* was macerated with ethanol for 8 days at room temperature. After extraction, the ethanolic solution was filtered and concentrated to dryness. The ethanol extract of *I. serrulata* was initially chromatographed on Sephadex LH-20 giving six sub fractions (Table S2). Fraction IsF2 exhibited the highest yield, accounting for 62.61% of the crude extract, whereas IsF6 showed the lowest value, corresponding to only 0.78%.

From fraction IsF5 (1.78% yield), two glycosylated aromatic constituents were detected and tentatively identified as tachioside (**1**) and isotachioside (**2**) based on comparative analysis of their ^1^H and ^13^C NMR spectroscopic data with literature reports (Table S1). Both constituents were present as a mixture in similar relative proportions, as inferred from the relative intensities of diagnostic signals observed in the ^1^H NMR spectrum.

The ^1^H NMR spectrum of the mixture displayed characteristic signals attributable to two closely related trisubstituted aromatic systems. Compound **1** showed signals at δ_H_ 6.69 (d, *J* = 8.7 Hz), 6.47 (d, *J* = 2.7 Hz) and 6.30 (dd, *J* = 8.7, 2.7 Hz), indicative of a trisubstituted aromatic ring. Its anomeric proton resonated at δ_H_ 4.74 (d, *J* = 7.3 Hz), consistent with a β-linked glucopyranosyl unit, and a methoxy group was observed as a singlet at δ_H_ 3.82 [22,23,24,25].

Compound **2** exhibited a similar aromatic proton pattern with resonances at δ_H_ 7.01 (d, *J* = 8.7 Hz), 6.80 (d, *J* = 2.7 Hz), and 6.58 (dd, *J* = 8.7, 2.7 Hz) were consistent with a 1,2,4-trisubstituted aromatic ring. An anomeric proton signal at δ_H_ 4.70 (d, *J* = 7.6 Hz) indicated the presence of a β-glucopyranosyl moiety, with the coupling constant supporting a β-configuration at the anomeric center. In addition, a methoxy group was evidenced by a singlet at δ_H_ 3.80 [22,24,26,27].

**Figure 1:**
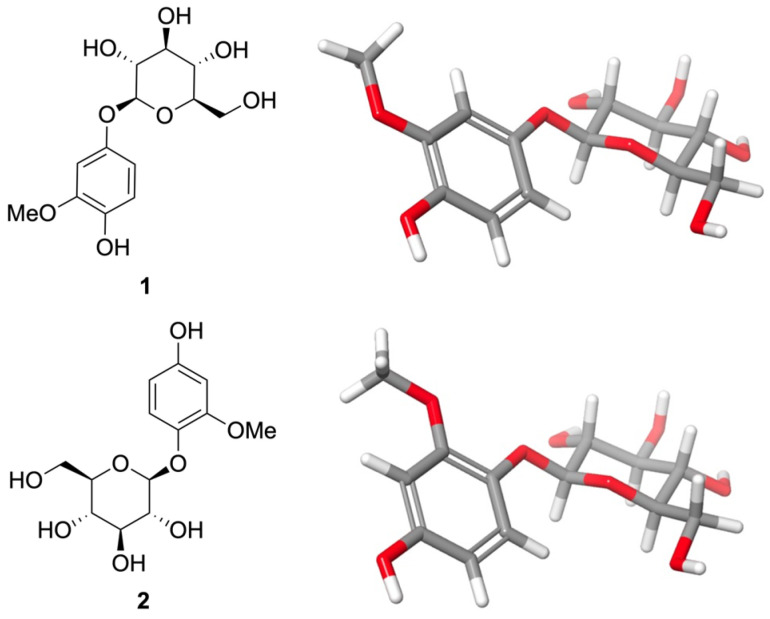
Chemical and minimized 3D structures of the glycosylated aromatic constituents inferred in *Isotachis serrulata* based on comparative ^1^H and ^13^C NMR spectroscopic data: tachioside (**1**) and isotachioside (**2**). Schrödinger Release 2024-4: Maestro v. 14.2.118, Schrödinger, LLC, New York, NY, USA.

The corresponding ^13^C NMR data further supported the presence of two methoxylated phenyl β-glucosides, showing resonances attributable to aromatic carbons, anomeric carbons, and sugar moieties in agreement with previously reported values for isotachioside and tachioside. Based on these spectroscopic features and literature comparison, the constituents detected in fraction IsF5 were tentatively assigned as tachioside (methoxyhydroquinone-4-β-D-glucopyranoside) (**1**) and isotachioside (methoxyhydroquinone-1-β-D-glucopyranoside) (**2**), Figure 1. Both constituents are consistent with data reported in the literature, which explains their similar chromatographic behavior on TLC and silica gel column chromatography. To the best of our knowledge, this appears to represent the first indication of the presence of both compounds in *I. serrulata*.

The antiproliferative activity of the crude ethanolic extract (IsEx) and its fractions (IsF1–IsF6) obtained from *I. serrulata* was evaluated against five human tumor cell lines, including A549 and SW1573 (non-small cell lung carcinoma), HBL-100 (mammary gland), HeLa (human cervical carcinoma), and T-47D (breast carcinoma). Cells were exposed to three concentrations of each sample (2.5, 25, and 125 µg/mL).

**Figure 2:**
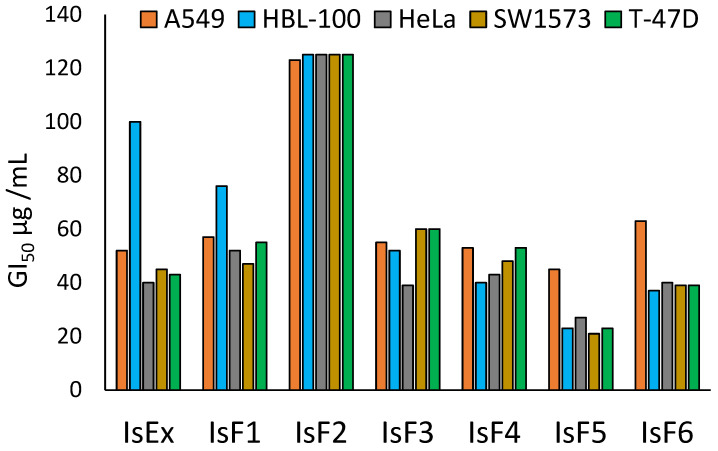
The antiproliferative activity (GI_50_ µg/mL) of crude extracts and fractions of liverwort *Isotachis serrulata*.

Antiproliferative effects were expressed as GI_50_ values in µg/mL, defined as the concentration required to inhibit 50% of cell growth, as summarized in Figure 2. According to the criteria established by the National Cancer Institute (NCI), samples with GI_50_ values lower than 20 µg/mL are considered active (A), those with GI_50_ values between 20 and 100 µg/mL are regarded as moderately active (MA), and those with GI_50_ values higher than 100 µg/mL are considered inactive (IA).

The crude ethanolic extract (IsEx) of *I. serrulata* exhibited moderate antiproliferative activity (MA) against all evaluated human tumor cell lines (Figure 2), with GI_50_ values ranging from 40 to 100 µg/mL. Similarly, fractions IsF1, IsF3, IsF4, IsF5, and IsF6 showed moderate activity across all tested cell lines.

Among the evaluated fractions, IsF5 displayed the most pronounced antiproliferative effects, yielding the lowest GI_50_ values, particularly against the SW1573 non-small cell lung cancer line (21 µg/mL), as well as the HBL-100 mammary gland (23 µg/mL) and T-47D breast cancer (23 µg/mL) cell lines. In contrast, fraction IsF2, which represented 62.61% yield of the extract, was classified as inactive (IA) against all tested cell lines, with GI_50_ values ranging from 123 to 125 µg/mL.

Fraction IsF5 was selected for further phytochemical investigation based not only on its comparatively lower GI_50_ values but also on its simpler and better-resolved TLC profile, which facilitated compound detection and structural analysis. Although IsF6 exhibited moderate antiproliferative activity, its lower yield and more complex chromatographic profile limited additional chemical characterization within the scope of the present study.

Several studies have documented cytotoxic effects of crude liverwort extracts against diverse cancer cell lines [28,29,30,31,32]. Similar antiproliferative and cytotoxic effects have been reported for liverwort-derived metabolites in other genera. For instance, prenylated bibenzyls isolated from *Radula apiculata* exhibited cytotoxic activity against human cancer cell lines, with reported IC_50_ values in the low micromolar range [33]. Likewise, bis-bibenzyl compounds obtained from *Pellia endiviifolia* have shown significant cytotoxic effects against various tumor cell lines, including glioblastoma and leukemia models, with IC_50_ values typically ranging from approximately 5 to 30 µM [34]. Although direct comparison is not possible due to differences in experimental conditions and activity metrics, these results are consistent with the moderate antiproliferative activity observed for the fractions of *I*. *serrulata* in the present study.

According to the criteria established by the National Cancer Institute (NCI), compounds with GI_50_ values ≤ 20 µg/mL are classified as active. Although none of the evaluated samples fully met this threshold, fraction IsF5 exhibited GI_50_ values approaching this limit, particularly against the SW1573 cell line. These results suggest that bioactive constituents are enriched within this fraction, indicating that IsF5 warrants further phytochemical investigation and pharmacological evaluation (Figure 2).

In contrast, fraction IsF2 was inactive (GI_50_ > 100 µg/mL) in all cell lines, indicating the absence or low concentration of antiproliferative constituents in this fraction. This clear distinction demonstrates the importance of fractionation in enhancing the biological activity of plant extracts and isolating specific active compounds. Fractions IsF1, IsF3, IsF4 and IsF6 exhibited moderate antiproliferative effects, with GI_50_ values between 39 and 76 µg/mL, less potent than IsF5.

Notably, the differential sensitivity observed among the various cell lines, which may be attributed to the intrinsic molecular characteristics of each cancer type. These findings support the need for additional studies aimed at elucidating the mechanism of action, selectivity, and in vivo efficacy of the most active fractions, particularly IsF5. The fraction IsF5 has the most promising antiproliferative profile among all tested fractions, especially against lung and breast derived cell lines. Further research is needed to identify and isolate the active compounds that are responsible for this effect.

The glycosylated aromatic constituents tentatively assigned as tachioside (**1**) and isotachioside (**2**) were detected as a mixture in subfraction 5 derived from IsF5, the fraction exhibiting the highest antiproliferative activity against the tested cancer cell lines. It should be noted that the isolated compounds were obtained as a mixture and could not be evaluated individually due to the limited amount of material. Therefore, a direct causal relationship between these constituents and the observed antiproliferative activity cannot be conclusively established. Nevertheless, the enrichment of these metabolites in the most active fraction (IsF5) suggests that they may contribute to the observed biological effects. Further studies involving the isolation of individual compounds and their biological evaluation will be necessary to confirm their specific activity.

The antiproliferative activity of liverwort extracts can be attributed to the presence of compounds such as bibenzyls, terpenoids, and prenylated flavonoids [31]. Although there are no direct reports on the anticancer activity of tachioside (**1**) and isotachioside (**2**) themselves, closely related phenolic glycosides and structurally similar compounds have been associated with relevant biological effects. For example, isotachioside and tachioside have been identified among phenolic glycosides in *Gentiana* and other plant species with notable antioxidant properties, which are often correlated with cytoprotective effects in cellular systems [35]. Additionally, phenylethanoid glycosides—an analogous class of compounds—have been shown to exhibit cytotoxic and antiproliferative actions against various cancer cell lines, as reported for acteoside and plantamajoside in multiple models (including breast and hepatocarcinoma cells) [36]. These findings, while indirect, suggest that the phenolic glycoside scaffold represented by compounds **1** and **2** merits further study in anticancer screens when adequate quantities become available.

From a synthetic perspective, both positional isomers could in principle be accessed through regioselective glycosylation of 2-methoxyhydroquinone derivatives. However, control of regioselectivity and stereochemistry in glycosylation reactions may require specific conditions. Future studies may explore synthetic strategies to obtain enough quantities of individual compounds for detailed biological evaluation and structure–activity relationship analysis.

### Species Distribution Modeling

**Figure 3:**
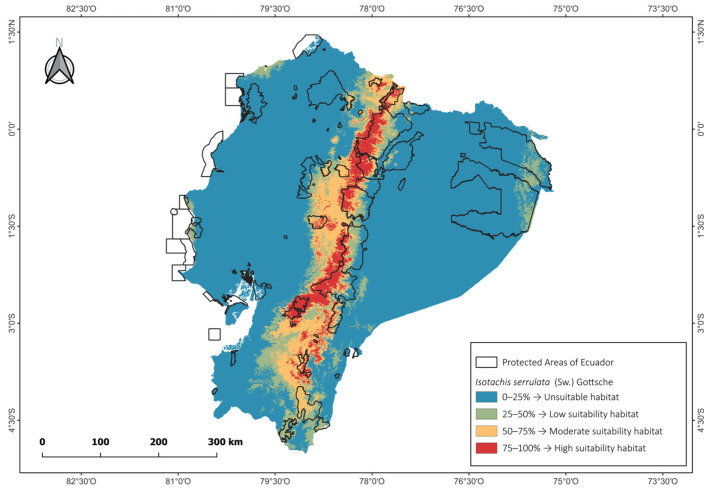
Geographical location and predicted potential distributions model of *Isotachis serrulata* obtained using MaxEnt models for current climatic conditions.

The potential distribution models indicated that *I. serrulata* is unevenly distributed along the Andean Cordillera of Ecuador, with higher suitability concentrated in the southern and central Andes. In particular, the species was well documented in the provinces of Loja and Zamora Chinchipe, while lower suitability and fewer occurrence records were predicted for the central and northern Andes (Figure 3).

The suitable habitat of *I. serrulata* was primarily associated with evergreen montane and lower montane forests of the southeastern Andes, including páramo ecosystems. This pattern is consistent with reports from tropical regions, where the species has been documented growing in montane tropical forests and páramo ecosystems [37,38,39,40]. These habitats are characterized by high humidity and specific climatic conditions that appear to favor the establishment and persistence of the species.

According to the MaxEnt model, elevation (65.2%), precipitation of the warmest quarter (20.7%), precipitation seasonality (8.6%) and mean diurnal range (4.8%) were identified as the most influential environmental variables limiting the potential distribution of *I. serrulata* in Ecuador. Model performance evaluation yielded an area under the curve (AUC) value of 0.911, indicating a good predictive model for *I. serrulata*.

The predicted potential distribution of *I. serrulata* along the Ecuadorian Andes follows a pattern consistent with the ecological preferences reported for montane liverwort species, which are strongly associated with humid environments, stable microclimatic conditions, and relatively narrow altitudinal ranges [41,42,43]. The concentration of suitable habitats in the southern, central and northern Andes (e.g., Loja, Zamora Chinchipe, Azuay, Pichincha and Carchi provinces). Accordingly, this agrees with floristic surveys indicating that bryophyte diversity in Ecuador is related with areas with high elevation and precipitation [44,45]. Similar distribution trends have been documented for other Andean liverwort taxa, where precipitation regimes and montane forest continuity play a decisive role in shaping species occurrence [46].

**Figure 4:**
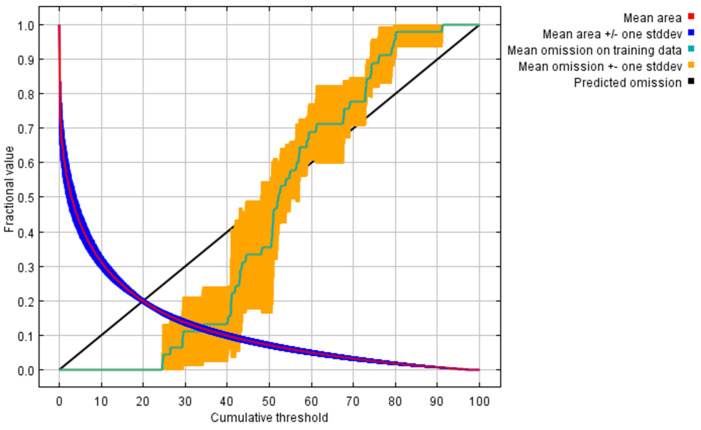
Omission rates versus predicted area for *Isotachis serrulata* obtained using MaxEnt models for current climatic conditions.

From a modeling perspective, the MaxEnt results highlight precipitation-related variables and elevation as the main limiting factors controlling the distribution of *I. serrulata*, a pattern frequently observed in ecological niche models of bryophytes and other plant species [47,48]. Precipitation seasonality and moisture availability related to elevation have been identified as key drivers influencing liverwort establishment, survival, and distribution [49]. The high AUC value obtained (0.867) indicates good model performance and is comparable to values reported in previous MaxEnt-based studies focusing on bryophyte and fern species in mountainous tropical regions [50,51,52]. This pattern is corroborated by the omission rate curve for *I. serrulata*, which closely follows the expected omission line (Figure 4), indicating good model calibration and robust predictive performance. The red line represents the mean predicted area, the black line corresponds to the predicted omission rate, and the light blue line indicates the omission rates for the training samples. These results support the robustness of the model and reinforce its usefulness for guiding future field surveys, conservation planning, and biogeographical studies of poorly documented liverwort species in the Andes.

## 3. Materials and Methods

### 3.1. Plant Material and Study Area

**Figure 5:**
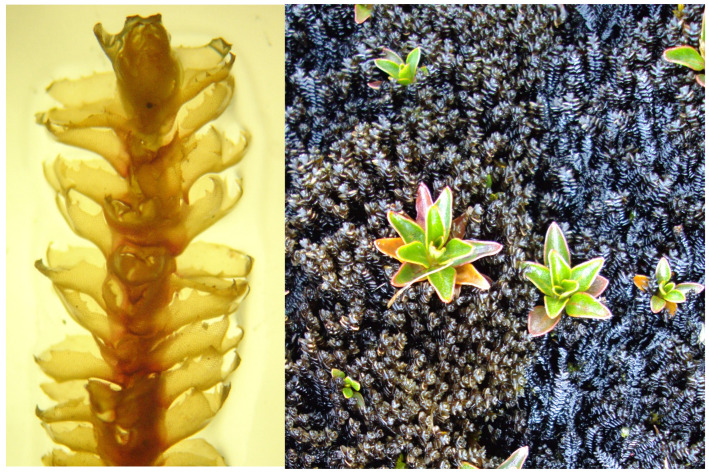
Representative photograph of *Isotachis serrulata* collected from its natural habitat in southern Ecuador.

*Isotachis serrulata* (Figure 5) was collected in southern Ecuador in October 2025, in montane Andean ecosystems characterized by humid climatic conditions Sampling sites were located at Zamora Chinchipe province (3°59′24″ S, 79°6′0″ W) at 2271 m a.s.l.

The plant material was taxonomically identified by curator of bryophytes and lichens at the HUPL Herbarium, and a voucher specimen (No. AB-1339) was deposited in the herbarium of the Universidad Técnica Particular de Loja (HUTPL)-Bryophytes and lichen collection, Ecuador. These occurrence records were subsequently used for species distribution modeling analyses.

### 3.2. Extraction and Fractionation Procedures

Plant material free of visible impurities was dried in a forced-air dehydrator at 30 °C for 48 h prior to extraction. The dried material of *I. serrulata* (680 g) was crushed and subjected to static maceration with ethanol (EtOH, 5 L) for 8 days at room temperature, with manual agitation twice daily.

After extraction, the ethanolic solution was filtered and concentrated to dryness under reduced pressure using a rotary evaporator (Büchi R-210, Flawil, Switzerland), yielding the crude ethanolic extract (13.28 g; 1.95% *w*/*w*).

An aliquot of the crude ethanolic extract (1.2 g) was dissolved in methanol (MeOH) and centrifuged at room temperature at 3500 rpm for 10 min [53]. The resulting supernatant was applied to a Sephadex LH-20 column (2.5 × 150 cm) and eluted with MeOH (100%), affording 27 fractions. Based on thin-layer chromatography (TLC) analysis, these fractions were combined into six pooled fractions (IsF1–IsF6). All fractions were weighed and stored at −20 °C for subsequent analyses [54,55].

Fraction IsF5 (21.30 mg) was subjected to further separation by open-column chromatography on silica gel (Si-G60, 70–230 mesh; Merck, Darmstadt, Germany). Elution with a mixture of ethyl acetate and methanol (8:2, *v*/*v*) afforded 36 subfractions, which were monitored by TLC and combined into five pooled fractions. Among them, pooled fraction 5 yielded a mixture enriched in two glycosylated aromatic constituents, tentatively identified as tachioside (**1**) and isotachioside (**2**) (Figure 1), present in approximately similar relative proportions as inferred from the relative intensities of diagnostic signals in the ^1^H NMR spectrum and by comparison of their ^1^H and ^13^C NMR spectroscopic data with literature reports. NMR spectra were recorded on a Bruker AVANCE 600 MHz spectrometer equipped (Billerica, MA, USA) with a 5 mm TCI inverse detection cryoprobe. Samples were dissolved in MeOD, and chemical shifts were reported in parts per million (ppm) relative to the residual solvent signals (δ_H_ 3.31 and δ_C_ 49.0 ppm). Standard Bruker pulse sequences were employed for all NMR experiments.

Tachioside (Methoxyhydroquinone-4-β-D-glucopyranoside) (**1**): ^1^H NMR (500 MHz, MeOD) δ_H_ 6.69 (d, *J* = 8.7 Hz, H-6), 6.47 (d, *J* = 2.7 Hz, H-3), 6.30 (dd, *J* = 8.7, 2.7 Hz, H-5), 3.82 (s, 3H, –OMe), 4.74 (d, *J* = 7.3 Hz, H-1′), 3.85 (dd, *J* = 12.0, 2.1 Hz, H-6′), 3.67 (m, H-6′), 3.32–3.46 (m, 4H, H-2′–H-5′). ^13^C NMR (126 MHz, MeOD) δ_C_ 151.41 (C-4), 147.88 (C-2), 141.52 (C-1), 114.65 (C-6), 108.60 (C-5), 102.34 (C-1′), 102.43 (C-3), 76.74 (C-3′), 76.39 (C-5′), 73.59 (C-2′), 70.16 (C-4′), 61.23 (C-6′), 55.04 (–OMe) (Figures S1–S4).

Isotachioside (Methoxyhydroquinone-1-β-D-glucopyranoside) (**2**): ^1^H NMR (500 MHz, MeOD) δ_H_ 7.01 (d, *J* = 8.7 Hz, H-6), 6.80 (d, *J* = 2.7 Hz, H-3), 6.58 (dd, *J* = 8.6, 2.7 Hz, H-5), 3.80 (s, 3H, –OMe), 4.70 (d, *J* = 7.7 Hz, H-1′), 3.89 (dd, *J* = 12.0, 2.1 Hz, H-6′), 3.70 (m, H-6′), 3.32–3.46 (m, 4H, H-2′–H-5′). ^13^C NMR (126 MHz, MeOD) δ_C_ 153.47 (C-4), 150.58 (C-2), 139.65 (C-1), 119.07 (C-6), 106.28 (C-5), 102.88 (C-1′), 100.46 (C-3), 76.69 (C-3′), 76.61 (C-5′), 73.65 (C-2′), 69.97 (C-4′), 61.13 (C-6′), 55.19 (–OMe) (Figures S1–S4).

### 3.3. Species Distribution Modeling

A total of 14 geographical occurrence records of *I. serrulata* (Table S3) were compiled from published literature and biodiversity databases, including the Global Biodiversity Information Facility (GBIF; accessed on 20 July 2025) [56] and the HUTPL herbarium.

Nineteen bioclimatic variables were obtained from the WorldClim database (accessed on 20 July 2025) [57] and used as environmental predictors for species distribution modeling. Collinearity among the 19 bioclimatic variables from WorldClim and elevation was evaluated using the variance inflation factor (VIF). Variables with VIF > 10 were excluded, resulting in seven variables retained for modeling: Mean diurnal range (BIO2), isothermality (BIO3), mean temperature of wettest quarter (BIO8), precipitation of wettest month (BIO13), precipitation of driest month (BIO14), precipitation seasonality (BIO15) and precipitation of warmest quarter (BIO18).

Species distribution modeling was performed using the Maximum Entropy algorithm implemented in MaxEnt software version 3.4.4., with 10 replicate runs to improve model robustness. For model calibration, 75% of the occurrence records were randomly selected as training data, while the remaining 25% were used for model validation. Model performance was evaluated using the area under the receiver operating characteristic curve (AUC). According to established criteria, AUC values below 0.7 indicate poor model performance, values between 0.7 and 0.8 indicate fair performance, values between 0.8 and 0.9 indicate good performance, and values above 0.9 indicate excellent predictive ability. The continuous habitat suitability output from MaxEnt (0–1) was rescaled to percentages and classified into four suitability classes: 0–25% (Unsuitable habitat) 25–50% (low suitability habitat), 50–75% (Moderate suitability habitat) and 75–100% (High suitability habitat)

### 3.4. Cancer Cell Growth Inhibition Assay

*Cell lines*: Cell used in this study were purchased from the ATCC or were donated to the group by partner institutions. For general purpose screening, we use the following human solid tumor cell lines: A549 and SW1573 (non-small cell lung), HBL-100 (Mammary gland), T-47D (breast), and HeLa (human cervical).

*Cell culture*: Cells grow in RPMI 1640 medium supplemented with 5% FBS and 2 mM glutamine. Incubation takes place at 37 °C, 5% CO_2_ and 95% relative humidity.

*Samples for testing*: The samples are resuspended in the appropriate volume of DMSO. The maximum concentration of stock solution is 10 mg/mL. If the amount of sample provided is not enough, diluted stock solutions are prepared. In any case, the maximum test concentration (C_max_) was 125 μg/mL.

*Antiproliferative assay*: The test follows our implementation of the NCI-60 protocol [58]. The maximum test dose is C_max_ and exposure time is 48 h. The results of the three dose assays were reported as GI_50_ (concentration required for 50% inhibition of cell growth). The assay results for the extract and fractions screened were separated into three categories: **A**, activity (GI_50_ < 20 μg/mL); **MA**, moderately active (20 ≤ GI_50_ ≤ 100 μg/mL); **IA**, inactive (GI_50_ > 100 μg/mL) [59]. GI_50_ values are for comparative prioritization of complex mixtures during primary screening.

## 4. Conclusions

This study presents an integrated chemical, biological, and biogeographical assessment of the liverwort *I. serrulata* from southern Ecuador. The ethanolic extract and its fractions showed moderate antiproliferative activity, with fraction IsF5 displaying the strongest effects, particularly against lung and breast cancer cell lines.

Phytochemical analysis of IsF5 led to the identification of two glycosylated aromatic compounds, tachioside (**1**) and isotachioside (**2**), reported for the first time in this species. These compounds were obtained as a mixture and could not be evaluated individually due to limited material; therefore, their specific contribution to the observed antiproliferative activity could not be established.

Ecological niche modeling indicated that the distribution of *I. serrulata* in Ecuador is mainly influenced by precipitation and elevation, with suitable habitats located in the northern, central and southern Andes. The performance of the MaxEnt model supports its potential usefulness for future field surveys, conservation, and bioprospecting efforts.

Overall, this work highlights the relevance of *I. serrulata* as an underexplored species combining phytochemical characterization, biological evaluation, and ecological modeling. The results should be considered preliminary, and further studies are required to isolate individual metabolites, evaluate their biological activity, and clarify possible structure–activity relationships.

## Data Availability

Data are available from the authors upon reasonable request.

## Supplementary Materials

The following supporting information can be downloaded at: https://www.mdpi.com/article/10.3390/molecules31071208/s1, Table S1: Comparison of selected ^1^H and ^13^C NMR data obtained from the mixture detected in subfraction 5 of IsF5 (MeOD) with literature values reported for tachioside and isotachioside; Table S2: The antiproliferative activity of crude ethanolic extract and fractions of liverwort Isotachis serrulata against human cancer cell lines; Table S3: Occurrence records of *Isotachis serrulata* used for species distribution modeling; Figure S1: ^1^H NMR spectrum (500 MHz, MeOD) of the mixture obtained from subfraction 5 of IsF5 containing tachioside and isotachioside; Figure S2: ^13^C NMR spectrum (126 MHz, MeOD) of the mixture obtained from subfraction 5 of IsF5 containing tachioside and isotachioside; Figure S3: HSQC spectrum (MeOD) of the mixture obtained from subfraction 5 of IsF5, showing distinct anomeric and aromatic ^1^H–^13^C correlations consistent with the presence of two glycosylated aromatic constituents; Figure S4: HMBC spectrum (MeOD) of the mixture obtained from subfraction 5 of IsF5, showing long-range correlations supporting the differentiation of two aromatic glucosides.
