# Supplementary material for: Evidence of Antiproliferative Activity in the Liverwort Isotachis serrulata from Southern Ecuador
Source: Molecules. 2026 Apr 6;31(7):1208. doi: 10.3390/molecules31071208 (PMC13074347; doi:10.3390/molecules31071208)

Supplementary Materials

Evidence of Antiproliferative Activity in the Liverwort *Isotachis serrulata* from Southern Ecuador

José Miguel Andrade [1,2], Ángel Benítez [3] , Aday González-Bakker [2], Luis Cartuche [4], José M. Padrón [2,] Ana R. Díaz-Marrero [5,6,*] and José J. Fernandez [2,6,]

[1] Departamento de Química, Universidad Técnica Particular de Loja (UTPL), Calle Paris s/n y Praga, 110107, Ecuador; jmandrade@utpl.edu.ec (J.M.A.)

[2] Instituto Universitario de Bio-Orgánica Antonio González (IUBO AG), Universidad de La Laguna (ULL), 38206 La Laguna, Tenerife, Spain; ; alu0101535456@ull.edu.es (J.M.A.); agonzaba@ull.es (A.G.-B.); jmpadron@ull.es (J.M.P.); jjfercas@ull.es (J.J.F)

[3] Biodiversidad de Ecosistemas Tropicales-BIETROP, Herbario HUTPL, Departamento de Ciencias Biológicas y Agropecuarias, Universidad Técnica Particular de Loja, San Cayetano s/n, 1101608 Loja, Ecuador; arbenitez@utpl.edu.ec (Á. B.)

[4] Facultad de Ciencias Agropecuarias. Universidad Técnica de Machala, 5.5 km Pan-American Av., Machala 070150, Ecuador, lcartuche1@utmachala.edu.ec (L.C.)

[5] Instituto de Productos Naturales y Agrobiología (IPNA), Consejo Superior de Investigaciones Científicas (CSIC), Avenida Astrofísico Francisco Sánchez 3, 38206 La Laguna, Tenerife; adiazmar@ipna.csic..es (A.R.D.)

[6] Biotecnología Marina, IUBO-ULL, Unidad Asociada al IPNA-CSIC, 38206 La Laguna, Tenerife, Spain

[7] Departamento de Química Orgánica, Universidad de La Laguna (ULL), 38206 La Laguna, Tenerife, Spain

* Correspondence: jmandrade@utpl.edu.ec

Table of Contents

1	**Table S1.** Comparison of selected ^{1}H and ^{13}C NMR data obtained from the mixture detected in subfraction 5 of IsF5 (MeOD) with literature values reported for tachioside and isotachioside.
2	**Table S2.** The antiproliferative activity of crude ethanolic extract and fractions of liverwort Isotachis serrulata against human cancer cell lines.
3	**Table S3.** Occurrence records of *Isotachis serrulata* used for species distribution modeling
4	**Figure S1.** ^{1}H NMR spectrum (500 MHz, MeOD) of the mixture obtained from subfraction 5 of IsF5 containing tachioside and isotachioside.
5	**Figure S2.** ^{13}C NMR spectrum (126 MHz, MeOD) of the mixture obtained from subfraction 5 of IsF5 containing tachioside and isotachioside.
6	**Figure S3.** HSQC spectrum (MeOD) of the mixture obtained from subfraction 5 of IsF5, showing distinct anomeric and aromatic ^{1}H–^{13}C correlations consistent with the presence of two glycosylated aromatic constituents.
7	**Figure S4.** HMBC spectrum (MeOD) of the mixture obtained from subfraction 5 of IsF5, showing long-range correlations supporting the differentiation of two aromatic glucosides.

In addition to ^1H and ^{13}C NMR data, HSQC and HMBC spectra were recorded for the mixture obtained from subfraction 5 of IsF5. Although signal overlap prevents unambiguous assignment of all correlations, distinct anomeric and aromatic ^1H–^{13}C correlations are observed, supporting the presence of two closely related glycosylated aromatic constituents and reinforcing the tentative assignment of tachioside and isotachioside. Based on comparison with literature data (Supplementary Figure S3 and Figure S4).

Table S1. Comparison of selected ^1H and ^{13}C NMR data obtained from the mixture detected in subfraction 5 of IsF5 (MeOD) with literature values reported for tachioside (**1**) and isotachioside (**2**).

	**1**		**Trung, B.V.; et al 2020**		**2**		**Trung, B.V.; et al 2020**	
**Position**	**δ^1 H, mult. (*J* in Hz)**	**δ^{13} C**	**δ^1 H, mult. (*J* in Hz)**	**δ^{13} C**	**δ^1 H, mult. (*J* in Hz)**	**δ^{13} C**	**δ^1 H, mult (*J* in Hz)**	**δ^{13} C**
1		141.5		143.0		139.7		141.1
2		147.9		149.3		150.6		152.1
3	6.47, d (2.7)	102.4	6.83, d (2.5)	104.9	6.80, d (2.7)	100.5	6.49, d (3.0)	101.9
4		151.4		152.8		153.5		154.9
5	6.30, dd (8.7, 2.7)	108.6	6.61, dd (8.5, 2.5)	110.1	6.58, dd (8.6, 2.7)	106.3	6.32, dd (8.5, 2.5)	107.7
6	6.69, d (8.7)	114.6	6.71, d (8.5)	116.0	7.01, d (8.7)	119.0	7.03, d (8.5)	120.6
3-OMe	3.82, s	55.0	3.85, s	56.4	3.80, s	55.2	3.83, s	56.6
1′	4.74, d (7.3)	102.3	4.76, d (7.5)	103.8	4.70, d (7.7)	102.9	4.72, d (7.5)	104.4
2′	3.32-3.46, m	73.6	3.43, dd (9.0, 7.5)	75.0	3.32-3.46, m	73.7	3.45, dd (9.0, 7.5)	75.1
3′	3.32-3.46, m	76.7	3.45, t	78.1	3.32-3.46, m	76.7	3.43, t	77.9
4′	3.32-3.46, m	70.2	3.37, t	71.6	3.32-3.46, m	69.9	3.70, t	71.4
5′	3.32-3.46, m	76.4	3.40, m	78.2	3.32-3.46, m	76.6	3.40, m	78.1
6′	3.85, dd (12.0, 2.1)<br>3.67, m	61.2	3.70, dd (11.5, 5.5)<br>3.92, dd (11.5, 2.5)	62.7	3.89, dd (12.0, 2.1)<br>3.70, m	61.1	3.70, dd (11.5, 5.5)<br>3.87, dd (11.5, 2.5)	62.6

^1H- NMR data for compounds **1**, **2** and literature (500 MHz), in MeOD

^{13}C- NMR data for compounds **1**, **2** (126 MHz), and literature (125 MHz), in MeOD

Table S2. The antiproliferative activity of crude ethanolic extract and fractions of liverwort *Isotachis serrulata* against human cancer cell lines. GI_{50} (μg/mL).

		Cancer cell lines									
		A549		HBL-100		HeLa		SW1573		T-47D	
Cmax	Sample	GI_{50}	NCI Criteria	GI_{50}	NCI Criteria	GI_{50}	NCI Criteria	GI_{50}	NCI Criteria	GI_{50}	NCI Criteria
125	Is-Ex	52	MA	100	IA	40	MA	45	MA	43	MA
125	Is-F1	57	MA	76	MA	52	MA	47	MA	55	MA
125	Is-F2	123	IA	125	IA	125	IA	125	IA	125	IA
125	Is-F3	55	MA	52	MA	39	MA	60	MA	60	MA
125	Is-F4	53	MA	40	MA	43	MA	48	MA	53	MA
125	Is-F5	45	MA	23	MA	27	MA	21	MA	23	MA
125	Is-F6	63	MA	37	MA	40	MA	39	MA	39	MA

Cmax: Maximum assay concentration (μg/mL). Exposure time 48 h.

Is-Ex: Ethanolic extract.

IsF1 – IsF5: Fractions obtained from ethanolic extract (Is-Ex).

NCI criteria rank: A, activity ($GI_{50} < 20$ μg/mL); MA, moderately active ($20 \leq GI_{50} \leq 100$ μg/mL); IA, inactive ($GI_{50} > 100$ μg/mL).

GI_{50}: Concentration (μg/mL required for 50% inhibition of cell growth).

Table S3. Occurrence records of *Isotachis serrulata* used for species distribution modeling

Species	Longitude	Latitude
Isotachis serrulata	-78.7666	-4.1166
Isotachis serrulata	-77.6869	0.5605
Isotachis serrulata	-78.5374	-3.9699
Isotachis serrulata	-79.1349	-3.9831
Isotachis serrulata	-79.156	-3.8218
Isotachis serrulata	-79.1349	-3.9831
Isotachis serrulata	-79.1356	-3.9909
Isotachis serrulata	-79.1176	-3.9891
Isotachis serrulata	-79.1065	-3.992
Isotachis serrulata	-79.1	-3.99
Isotachis serrulata	-79.0211	-4.0111
Isotachis serrulata	-79.0213	-4.011
Isotachis serrulata	-79.0581	-3.5992
Isotachis serrulata	-79.2224	-2.7824

Figure S1. ¹H NMR spectrum (500 MHz, MeOD) of the mixture obtained from subfraction 5 of IsF5 containing tachioside and isotachioside.

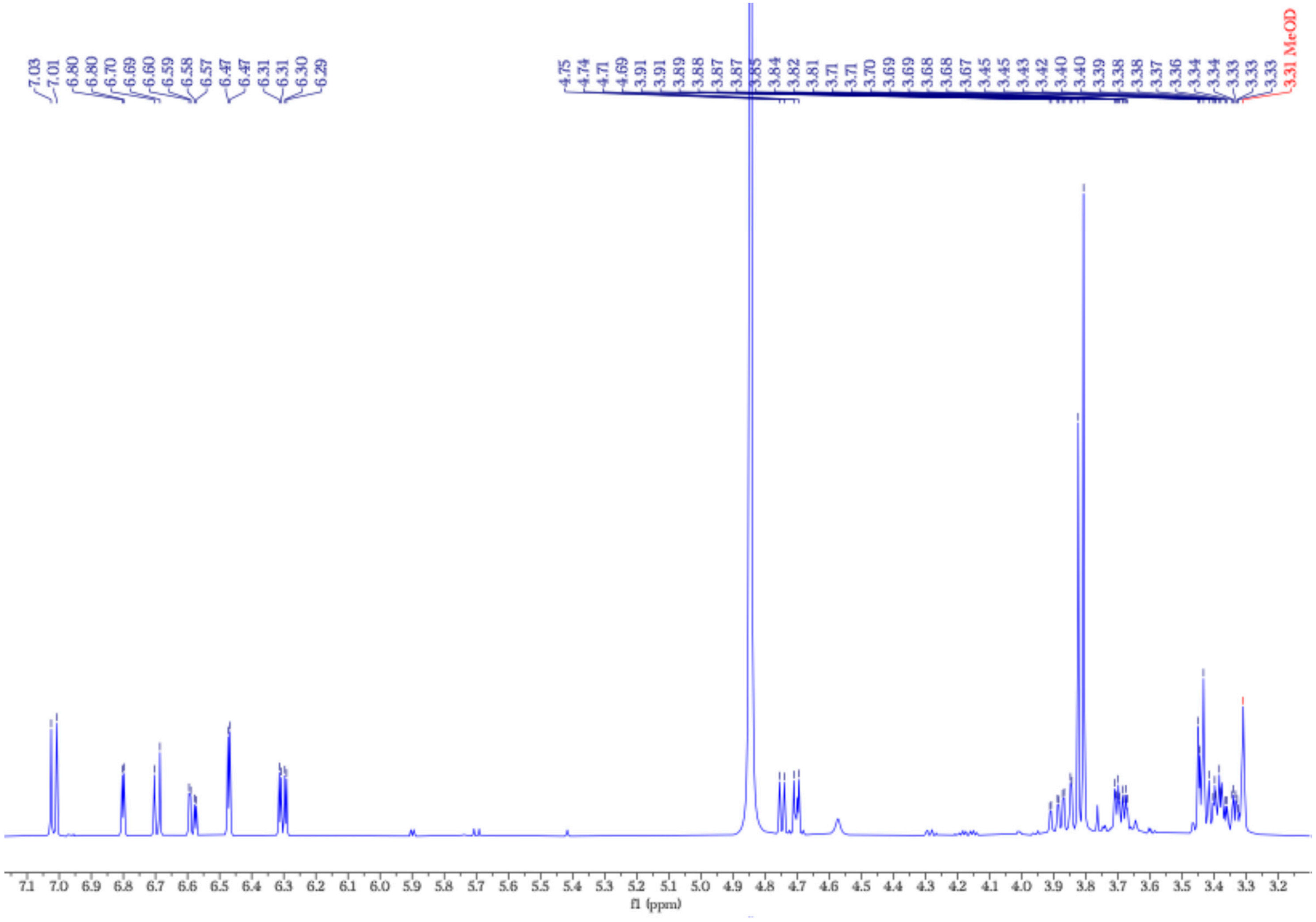

Figure S2. ^{13}C NMR spectrum (126 MHz, MeOD) of the mixture obtained from subfraction 5 of IsF5 containing tachioside and isotachioside.

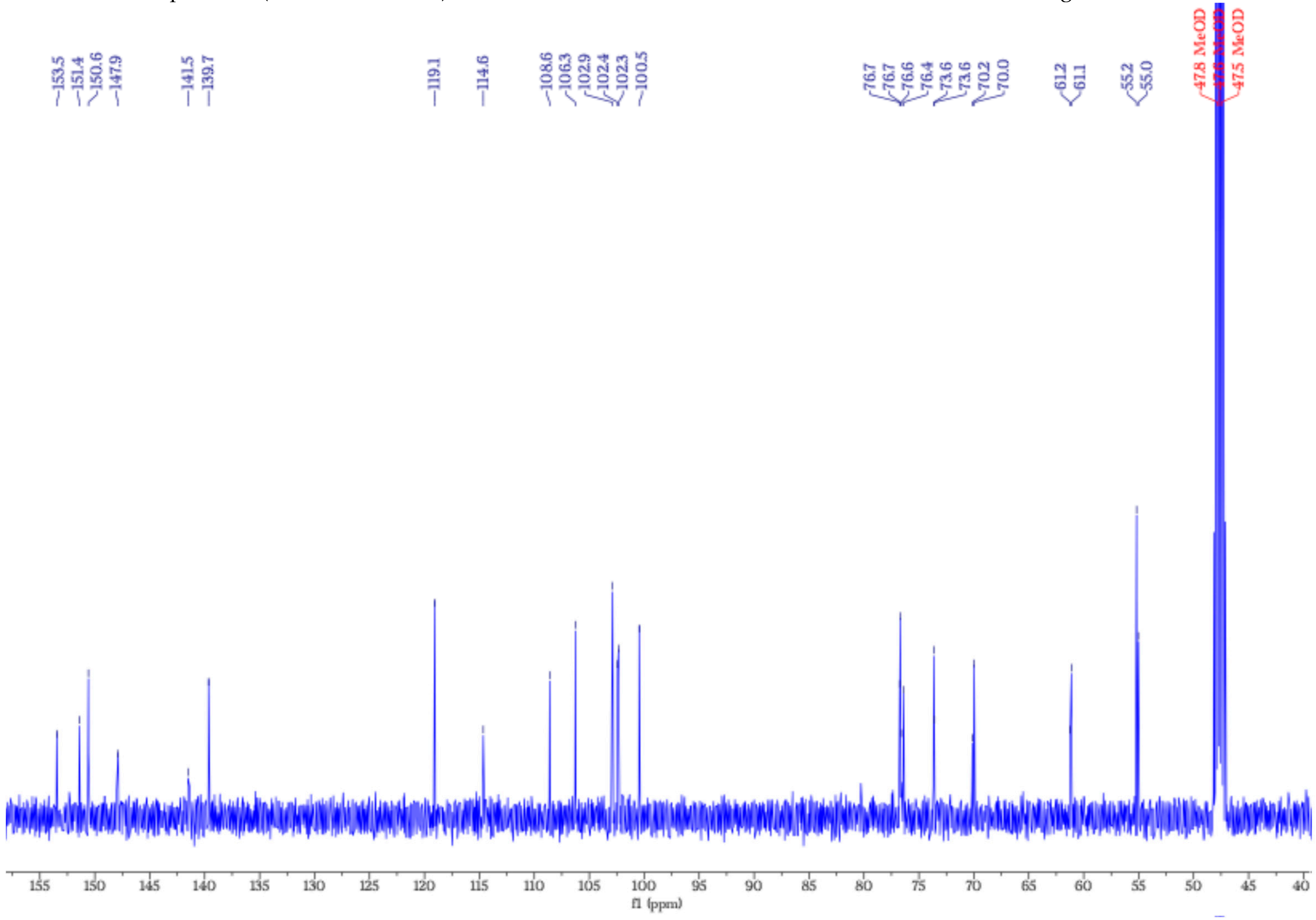

Figure S3. HSQC spectrum (MeOD) of the mixture obtained from subfraction 5 of IsF5, showing distinct anomeric and aromatic ^{1}H–^{13}C correlations consistent with the presence of two glycosylated aromatic constituents.

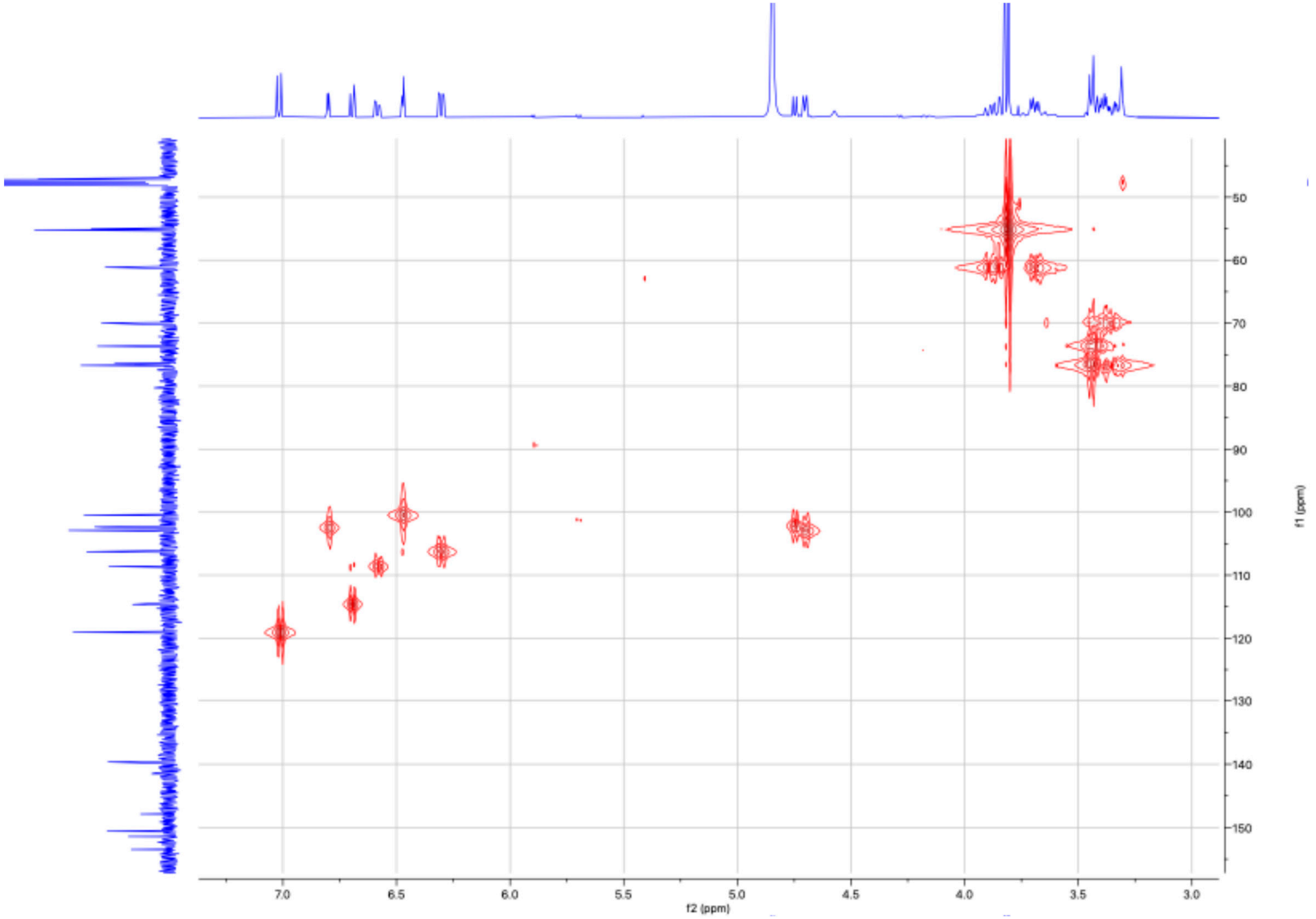

Figure S4. HMBC spectrum (MeOD) of the mixture obtained from subfraction 5 of IsF5, showing long-range correlations supporting the differentiation of two aromatic glucosides.

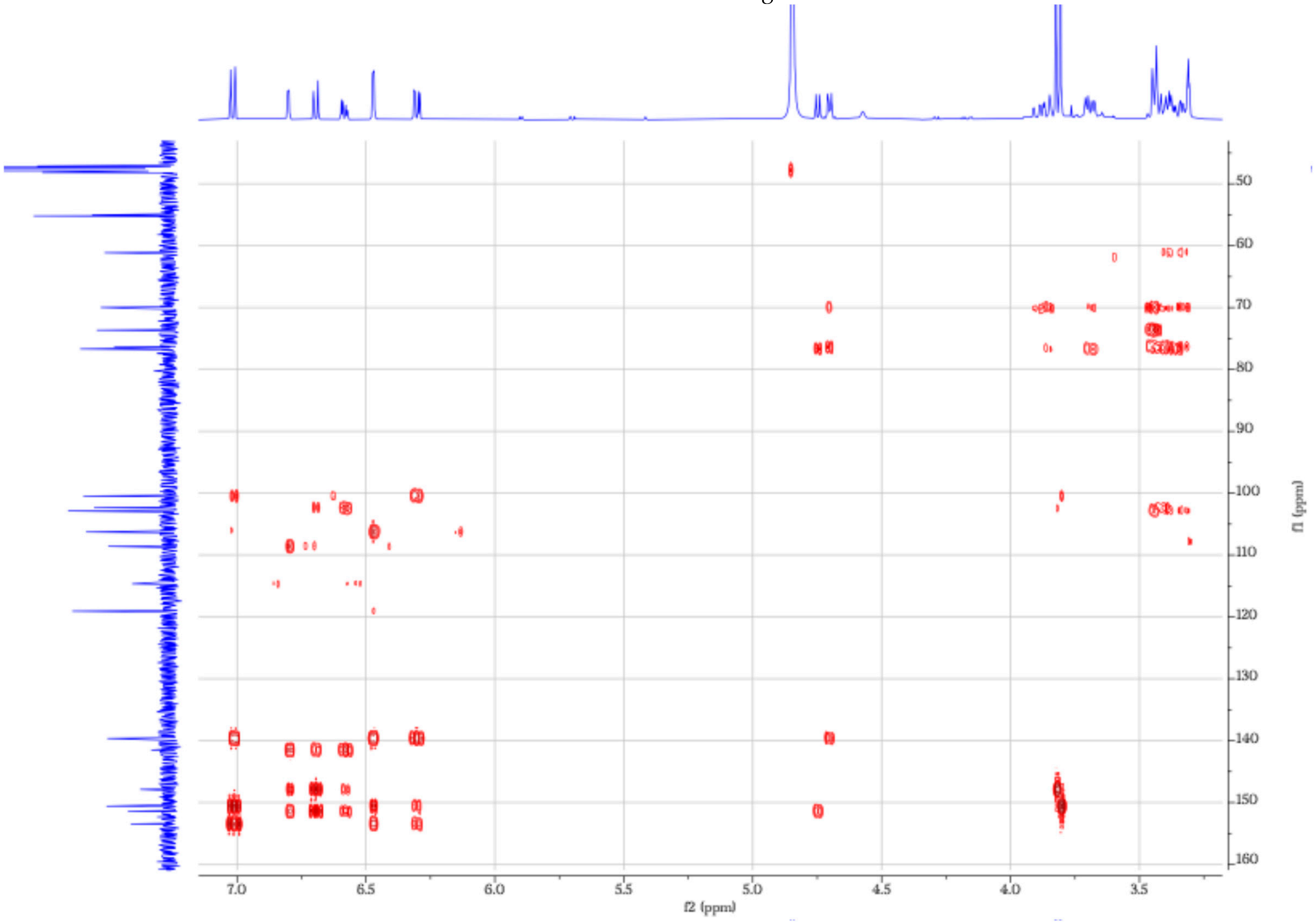

Supplement: Supplementary file 1 [file molecules-31-01208-s001.zip › molecules-4155858-supplementary-update.pdf]
